# Is the Combination of Insecticide and Mating Disruption Synergistic or Additive in Lightbrown Apple Moth, *Epiphyas postvittana*?

**DOI:** 10.1371/journal.pone.0160710

**Published:** 2016-08-08

**Authors:** David M. Suckling, Greg Baker, Latif Salehi, Bill Woods

**Affiliations:** 1 The New Zealand Institute for Plant & Food Research Limited, Christchurch, New Zealand and School of Biological Sciences, University of Auckland, Building 733, Tamaki Campus, Auckland, New Zealand; 2 Plant Biosecurity Cooperative Research Centre, Bruce ACT, Australia; 3 Entomology Unit, South Australian Research and Development Institute, Adelaide, SA, Australia; 4 Department of Food and Agriculture, South Perth, WA, Australia; 5 Better Border Biosecurity, Christchurch, New Zealand; USDA-ARS Beltsville Agricultural Research Center, UNITED STATES

## Abstract

Pest suppression from combinations of tactics is fundamental to pest management and eradication. Interactions may occur among tactical combinations and affect suppression. The best case is synergistic, where suppression from a combination is greater than the sum of effects from single tactics (AB >> A+B). We explored how mating disruption and insecticide interacted at field scale, additively or synergistically. Use of a pheromone delivery formulation (SPLAT^™^) as either a mating disruption treatment (i.e. a two-component pheromone alone) or as a lure and kill treatment (i.e. the two-component pheromone plus a permethrin insecticide) was compared for efficacy against the lightbrown apple moth *Epiphyas postvittana*. Next, four point-source densities of the SPLAT^™^ formulations were compared for communication disruption. Finally, the mating disruption and lure and kill treatments were applied with a broadcast insecticide. Population assessment used virgin female traps and synthetic pheromone in replicated 9-ha vineyard plots compared with untreated controls and insecticide-treated plots, to investigate interactions. Lure and kill and mating disruption provided equivalent suppression; no additional benefit accrued from including permethrin with the pheromone suggesting lack of contact. The highest point-source density tested (625/ha) was most effective. The insect growth regulator methoxyfenoxide applied by broadcast application lowered pest prevalence by 70% for the first ten weeks compared to pre-trial. Pheromone addition suppressed the pest further by an estimated 92.5%, for overall suppression of 97.7% from the treatment combination of insecticide plus mating disruption. This was close to that expected for an additive model of interactivity between insecticide and mating disruption (AB = A+B) estimated from plots with single tactics as 98% suppression in a combination. The results indicate the need to examine other tactical combinations to achieve the potential cost-efficiencies of synergistic interactions.

## Introduction

Range expansion is evident in many arthropod pests, as a result of globalisation, and consequently there is a rise in the number of eradication programmes started by governments [1]. Invasive insects can affect agricultural productivity, forest resources, human health and a wide range of natural ecosystem services [2,3]. Particular pests vary in degree of invasiveness, but one of the key factors affecting establishment, apart from propagule pressure, is the rate of population growth at low density during colonisation. A key phenomenon, termed the Allee effect, relates to the change in population growth rate as a function of density, and it is postulated that populations below a certain threshold will decline to extinction [4,5]. In fact, during an incursion of an unwanted exotic species, weak mate finding can be a barrier to establishment, which has potential to be exploited [6]. Coincidentally, several pest management tactics reduce mate location ability, and can create new opportunities for pest suppression, for example, using the sterile insect technique, mating disruption or male removal [7]. Pest suppression from combinations of management tactics is fundamental to both integrated pest management and eradication. However, it is important to know what interactions may occur among tactical combinations, to improve suppression and avoid redundancy [5,8]. Specific combinations of tactics such as mating disruption and insecticides can offer a beneficial bioeconomic synergy according to models [9] but field evidence is rare. The mechanisms involved with pheromones can directly affect the outcome whether density dependent or independent [10].

Certain pest orders and families have received attention for both pest impacts and the establishment of government-sanctioned eradication programmes, and the pest Tortricidae appear prominently amongst the Lepidoptera in both areas [11]. Government eradication programmes reported in the Global Eradication and Response database (http://b3.net.nz/gerda) have targeted a few of these including *Lobesia botrana* Den. & Schiff. recently established in California and Chile)[12], and the false codling moth (*Thaumatotibia leucotreta*, a major citrus pest in South Africa) [13].

*Epiphyas postvittana* (Walker), lightbrown apple moth (LBAM) is an indigenous Australian leafroller species which has become a pest of a range of orchard (e.g. pome fruit, citrus, stone fruit) and vineyard crops in several countries[14]. Larvae present a market access risk for exports of fruit commodities such as citrus and pome fruit, and its recent establishment in California was accompanied by a major pheromone trapping programme to delimit the pest, which spread rapidly in part because of human movement of plant commodities [15,16].

Mating disruption has had limited use as an eradication tactic against pest Lepidoptera [11]. Mating disruption has been long developed against LBAM [17,18], and a four-species pheromone blend is now used in commercial orchards in New Zealand [19]. Mating disruption technologies have been tested against LBAM in vineyards [20,21], apple [18] and citrus [22] orchards, pine forests [23] and urban areas [24], with ground and aerial releases of a range of formulations that have included polyethylene tubing, micro-encapsulated sprayables, SPLAT^™^ [23] and aerosols [25].

A range of attributes have been noted as important for mass trapping or lure and kill systems and can involve the use of female sex pheromone, with a persistent insecticide but contact with the insecticide is essential to efficacy with this tactic. [26,27]. A lure and kill system with a soft gel type formulation originally developed for codling moth was adapted to include LBAM, creating a formulation with two species as targets, with evidence for the insects contacting fatal droplets [28,29]. An experimental design where small exclusion cages prevented insect exposure to fatal dollops demonstrated the importance of high density point sources releasing high purity pheromone acting as false trails, resulting in more than 50% disruption of trap catch compared with controls, without the toxin. With the toxin available, the orchard population was reduced by 96% in 0.3 ha plots with 500 points/ha. Therefore, we considered it necessary to re-examine the effect of lure and kill with a different formulation.

Recent advances in the formulation of pheromone products have resulted in products which are well suited to rapid, mechanized, large-scale application from ground or air [30]. Field studies comparing the efficacy of a range of formulations for lightbrown apple moth suppression found that one of the newer products–SPLAT^™^ LBAM (ISCA Technologies, Riverside, California, USA)—performed better than the other tested products [20,23]. Whether with the limited incorporation of an insecticide (lure and kill; L&K) or without insecticides (mating disruption; MD), this technology offers a means of replacing older pest suppression technologies such as broad-spectrum insecticides, which lack target specificity and have potential for food contamination [31,32].

The behavioural mechanism of operation of lure and kill and mating disruption systems the same for both technologies in the case of fully attractive blends where false trails are produced, but attractive blends are not necessary or even always used in mating disruption [10,20]. It could be expected that the lure and kill droplets for LBAM would operate by male removal, since a previously-developed formulation did so [28], and also that increasing point-source density would increase efficacy [21]. Attractive synthetic sources enable the mechanism of point source competition with calling wild females, which is purported to provide density dependent suppression [10]. Furthermore, the purported inverse density-dependence of mating disruption might be expected to work best when combined with other tactics to generate better suppression than achieved using single tactics alone[8].

Theoretical population dynamics models underpin knowledge of how different tactics operate [33–35], but rarely if ever to our knowledge have field trials of mating disruption examined population changes from combinations of treatments where the treatment effects were isolated and compared together in combination.

However, eradications frequently seek multiple tactics, and specific combinations of interest have the potential to be cost effective at suppression, so knowledge of combinations of tactics is desirable, but more importantly, the underlying principles of how pheromone and insecticide tactics interact have not been elucidated previously, whether additive or synergistic in behaviour.

The feasibility of developing mating disruption or lure and kill as cost-effective tactics to be used in an integrated programme with other compatible technologies to suppress LBAM was investigated in three field experiments in South Australian vineyards. To decide whether lure and kill was worth pursing further, the relative efficacy of a) mating disruption compared with b) lure and kill formulations of the LBAM pheromone produced by ISCA Technologies (SPLAT^™^ HD) was assessed and compared with a c) positive control, the commercial standard pheromone treatment (Isomate^®^ LBAM) [22,36] and d) untreated negative control plots. A second experiment examined the attractiveness of the formulations, which is critical to understand the mechanisms involved. The effect of point-source density of the mating disruption product SPLAT^™^ HD LBAM was assessed in a third experiment. In a fourth experiment, operated in larger (9-ha) plots, we examined the effect of the mating disruption product SPLAT^™^ HD LBAM used in combination with a broadcast application of an insecticide (methoxyfenozide), by comparing with untreated controls over two generations, to determine the longevity of suppression. The results from the experiments were combined to determine whether the results from insecticide plus pheromone were simply additive (as hypothesised).

## Materials and Methods

These field experiments were conducted with Australian Pesticide and Veterinary Medicines Authority permits 11901, 12337 and 12973 respectively. In all experiments, pre- and post-treatment counts of male LBAM moths captured in red delta pheromone traps [37] were recorded weekly. Each trap was fitted with a standard rubber septa lure (Etec Crop Solutions Limited, Auckland, New Zealand) loaded with 3 mg of a blend of the two major LBAM pheromone components (95% (*E*)-11-tetradecenyl acetate (*E*11-14ac) and 5% (*E*)-9, (*E*)-11-tetradecadien-1yl acetate (*E* 9*E*11-14ac)) [38]. New lures were fitted every 12 weeks. Virgin female moths from a laboratory colony were used for comparison when available, although it was known that the 3 mg synthetic two component lures were more powerful and reliable [23].

### Experimental sites

The experiments were all conducted in the extensive and largely homogeneous vineyard plantings of Pernod Ricard Winemakers at Langhorne Creek, South Australia (-35.2967, 139.0172). These vineyards consisted of trellised rows with 2.5 m spacing aligned in an approximately E-W direction. The wire trellises were supported with panel posts of 1.8 m height spaced at 5.4 m intervals along the row. The traps were suspended on wires in the vineyard approximately 1.0 m above ground level

### Experiment 1: Relative efficacy of mating disruption and lure and kill

A randomized block design of 16 1.0-ha square plots with non-contiguous perimeters in a 32 ha area of Chardonnay vines was used to evaluate the relative efficacy of mating disruption and lure and kill formulations of the LBAM pheromone against the positive control of commercial standard pheromone treatment (Isomate^®^ LBAM) and an untreated negative control. The treatments were designed to test similar dosages of active pheromone constituents (four replicates each): (i) Isomate^®^ LBAM Plus Pheromone applied at the registered rate of 500 twist dispensers ha^-1^ tied to panel wires (81.6 g of *E*-11-teradecenyl acetate (*E*-11) and 3.4 g of *E*,*E*-9-11-tetradecadien-1-yl-acetate ha^-1^(*E*,*E*-9-11)); (ii) SPLAT^™^ HD LBAM (mating disruption treatment) applied as 1.0 g dollops to 740 panel posts ha^-1^ (70.3 g of *E*-11 and 3.7 g of *E*,*E*-9-11 ha^-1^); (iii) SPLAT^™^ HD LBAM plus 5% permethrin (lure and kill treatment) applied as 1.0 g dollops to 740 panel posts ha^-1^ (66.8 g of *E*-11 and 3.5 g of *E*,*E*-9-11 ha^-1^); and (iv) an untreated control. The treatments were applied on 22–23 March 2010 at approximately 1.5 m height to panel wires (ties) or posts (dollops). However, post application the mean dollop weight of the applied SPLAT^™^ products was estimated to be 0.84 g. As a result of this under-dosing, the actual application rate of the primary active ingredient in the SPLAT^™^ mating disruption and lure and kill products was respectively about 72.2% (58.9 g ha^-1^) and 68.8% (56.1 g ha^-1^) of the rate in the standard Isomate^®^ treatment. Red delta pheromone traps [37] and red rubber septa loaded with 3 mg of a binary blend of the LBAM pheromone components (95% (*E*)-11-tetradecenyl acetate (*E*11-14ac) and 5% (*E*)-9, (*E*)-11-tetradecadienyl acetate (*E* 9*E*11-14ac)) [38] were sourced from Etec Crop Solutions Limited, Auckland, New Zealand. Seven pheromone traps were placed in each plot evenly spaced (approximately 16.7 m apart) along a central 100 m row transecting the 1.0 ha plot area. Each trap was fitted with a septa and suspended on cordon wires in the vineyard at approximately 1.0 m above ground level. IN addition, counts were recorded weekly of male LBAM moths captured in red delta traps baited with 3 virgin-females (two traps per plot for 2 weeks pre-treatment and five traps per plot for 7 weeks post-treatment, limited by insect supply). These traps, which were displaced 5 m from traps with synthetic lures, were provisioned with a water source and three one-day-old virgin LBAM females weekly; mid-weekly any dead females were replaced with live one-day-old females.

### Experiment 2: Relative attractiveness of point sources of SPLAT^™^ HD LBAM

A 4x4 Latin square design with 30 m spacing between each trap was used to test the attraction to delta traps of four lures: rubber septa (3 mg), virgin females (5) and the two SPLAT treatments (MD and L&K). The traps (four replicates per treatment) were operated from 8 April 2010 to 26 May 2010 (49 days). SPLAT^™^ HD LBAM (mating disruption treatment) was applied as above 1.0 g dollops into traps (70.3 g of *E*-11 and 3.7 g of *E*,*E*-9-11 ha^-1^); SPLAT^™^ HD LBAM plus 5% permethrin (lure and kill treatment) was similarly applied as 1.0 g dollops into traps. Five virgin females per trap were used to raise the catch from this treatment after poor attractancy from three virgin females was seen in Experiment 1, and the data were corrected to estimate per female effects.

### Experiment 3: Point-source density effect on mating disruption from SPLAT^™^ HD LBAM

A five-replicate randomized-block design of 25 1.0-ha square plots with non-contiguous perimeters deployed across a 68 ha area of Chardonnay, Merlot and Cabernet Sauvignon vines at Langhorne Creek was used to evaluate five rates (0, 100, 225, 400 and 625 g ha^-1^) of the MD product SPLAT^™^ HD LBAM, selected on its performance in Experiment 1. The SPLAT^™^ was applied on 22–23 November 2010 to replicates 1–3 and on 13 December 2010 to replicates 4–5 as approximately 1 g dollops to panel posts at 1.5 m height, in the following pattern: (i) 100 dollops ha^-1^ applied in every fourth row to every second post; (ii) 225 dollops ha^-1^ applied in every second row to every second post and an additional 25 random dollops applied across each plot; (iii) 400 dollops ha^-1^ applied in every second row to every post; and (iv) 625 dollops ha^-1^ applied to 15 posts in every row in a ‘treat three posts, leave one untreated’ repeat sequence and an additional 25 random dollops applied across each plot. The two synthetic pheromone traps in each plot were checked and the catch of LBAM male moths scored weekly for eight weeks, and then the 15 plots for which the cumulative catch was relatively high and similar were chosen, allocated to each of three replicates (reps 1–3) based on spatial proximity, and within each replicate the five treatments were randomly allocated. Traps were similarly placed in a further 16 1.0 ha plots on 14 November 2010, checked weekly for four weeks, and then 10 plots similarly chosen and allocated to another two replicates (reps 4 and 5) and treatments again randomly allocated within each replicate block.

An Avatar^™^ (300 g indoxacarb kg^-1^) spray for LBAM control was applied by vineyard staff on 24 December 2010 to three blocks within the trial area (replicates 1 and 2 of the untreated control and replicate 1 of the 625 dollops ha^-1^ SPLAT treatment), which was not planned by the research team. A separate analysis was conducted to determine the effects.

### Experiment 4: Combination of insecticide and mating disruption technologies

A randomized block design of nine 9.0-ha square plots was used to evaluate the following three treatments (three replicates each): (i) a single tactic consisting of spray of the ecdysone receptor agonist insecticide Prodigy^®^ (240 g methoxyfenoxide L^-1^) applied at the registered rate of 250 ml product per 10^3^ L water ha^-1^ on 7–8 November 2011 to coincide with LBAM egg hatch and young larval development; (ii) dual tactics consisting of the same insecticide application plus SPLAT^™^ LBAM HD applied in a uniform pattern (as per Experiment 3) of 625 x 1-g dollops ha^-1^ on two occasions 11 weeks apart (28–29 November 2011 and 13–15 February 2012); and (iii) an untreated control.

Nine synthetic pheromone traps were placed centrally in each 9.0 ha plot in a 3 x 3 grid pattern. Three traps were placed on each of rows 50, 60 and 70 of the 120-row plots (2.5 m row spacing) and within each of these rows positioned on panels 20, 30 and 40 of the 56-panel rows (each panel 5.4 m length). The traps were assessed and serviced weekly for seven weeks prior to the insecticide treatment application (i.e. 9 September to 3 November 2011), three weeks between the insecticide application and the first of the SPLAT^™^ applications, 10 weeks between the first and second SPLAT^™^ applications (i.e. 30 November 2011 to 9 February 2012) and ten weeks following the second SPLAT^™^ application (i.e 16 February to 26 April 2012). In addition, 36 virgin female-baited traps (1 female per trap) were deployed ~5m from synthetic pheromone traps on the same layout as above, with four traps per replicate, weekly for 17 weeks. The entry-only traps were cylindrical, approximately 180 mm length and 100 mm diameter, and constructed from a plastic PET bottle fitted with tapered fly-wire mesh funnel ends with a 7 mm aperture for the males to enter. Single virgin female moths were released weekly into traps and females were dissected for mating.

### Data analysis

For Experiments 1 and 3, ANOVA models on transformed (ln(x+1)) pre-treatment and post-treatment pheromone trap and virgin female trap data were performed using GenStat. Means were separated using Tukey tests (α = 0.05). In the analysis of Experiment 1, treatment, trap position (end of transect or middle position) and post-treatment time interval were included as factors. For the final analysis, the model was performed without the control and the outer trap (traps 1 and 7) data, and with three time intervals (post-treatment weeks 1–3, 4–6 and 7–10) as repeated measures. In the analysis of Experiment 3, the outer trap data were excluded and exponential regression models were fitted. Data in all cases were back-transformed for presentation.

A Disruption Index (DI) was calculated for pheromone trap data in experiments 1, 3 and 4 as [(1-(number of males per treatment trap/number of males per control trap))*100][39]. For Experiments 1 and 3, the outer traps were excluded from these calculations. The resultant percentages were angular [arcsin(square-root(x))] transformed, and analysed using ANOVA, with the means separated using Tukey tests (α = 0.05). In practice, the product of suppression from two tactics is calculated as the product of the two survivorships and converted back to suppression (1-survivorship). This was compared with the suppression expected from an additive model (AB = A + B), as well as a synergistic model (AB > A + B).

## Results

### Experiment 1: Relative efficacy of mating disruption and lure and kill

The trap catches were similar in all plots in the pre-treatment period (6 weeks) (F _3, 9_ = 0.97; *P* = 0.450) (Table 1). During the 10-week post-treatment period (22–23 March to 2 June 2010) the numbers of moths captured in the pheromone traps in the control plots steadily increased from a mean of 0.09 to 2.10 moths per trap per day. There was a strong trap position effect in the pheromone-treated plots, with the outer pheromone traps positioned at each end of the transect in each plot catching significantly higher numbers of moths (F_6, 54_ = 23.65; *P* < 0.001). Hence the data for the outer traps were excluded from the rest of the post-treatment analyses. This effect was likely to be the result of the reduced trap competition in the outer semicircle around the end traps, which is also evident in the corner traps in square arrays [40].

**Table 1:** Pheromone rates, point source densities and synthetic pheromone trap catches of *Epiphyas postvittana* (lightbrown apple moth) in a comparison of mating disruption with lure and kill over 10 weeks in a commercial vineyard, Langhorne Creek, South Australia. The positive control was Isomate^®^ LBAM.

Treatment	Pheromone amount per dispenser or dollop (mg)	Source pointsper ha	Active ingredient g/ha	Males per trap per day(mean ± SE)^a^		DI^b^^,^^c^
				Pre-treatment	Post-treatment	
Untreated	0	0	0	0.023 ± 0.006 A	1.376 ± 0.100 A	-
Isomate^**®**^ LBAM	170.0	500	85.0	0.041 ± 0.010 A	0.052 ± 0.008 Ba	96.0 A
SPLAT^™^ MD	83.8	740	62.0	0.018 ± 0.005 A	0.109 ± 0.022 Ba	91.3 A
SPLAT +insecticide	79.7	740	59.0	0.024 ± 0.006 A	0.130 ± 0.033 Ba	89.2 A

^*a*^ Mean of Traps 2–6 (Traps 1 and 7 excluded).

^*b*^ Different letters within a column indicate significant differences among treatments (P≤0.05; Tukey test). For the Post-treatment column, the upper case letters apply to the ANOVA with the control, and the lower case letters apply to the ANOVA that excluded the control.

^*c*^ DI = (1-(no. males per treatment trap/no. males per control trap)) x 100.

**Fig 1:**
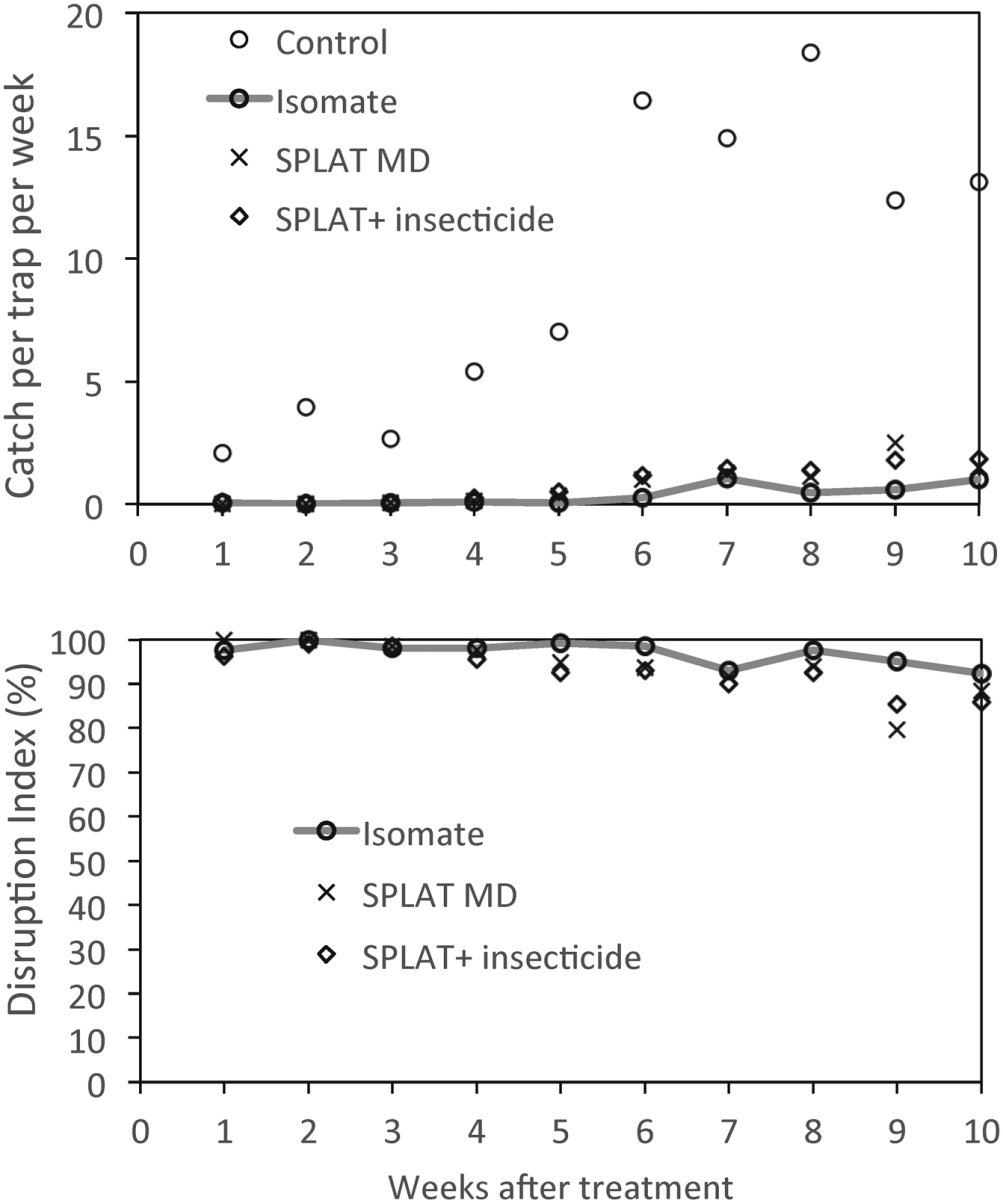
Mean daily catch per trap per week of *Epiphyas postvittana* in monitoring traps in a commercial vineyard, Langhorne Creek, South Australia (top) and impact of mating disruption (MD; Isomate^®^ LBAM and SPLAT^™^ LBAM), compared with untreated controls and SPLAT^™^ LBAM with insecticide (permethrin).

For the 10-week post-treatment assessment period, synthetic sex pheromone trap catches were significantly lower in the plots treated with a pheromone treatment, irrespective of treatment type, than the catches in the untreated control plots (*F*_*2*,*6*_ = 5.41; *P* = 0.045) (Table 1 and Fig 1). With the untreated control data omitted from the analysis, the pheromone trap catch was not significantly different between the three pheromone treatments (*F*_*2*,*6*_ = 2.38; *P* = 0.174) (Table 1). However, this treatment effect was dependent on the post-treatment time period examined (*F*_*2*,*90*_ = 151.9; *P* < 0.001) (Table 2). Trap catches were similar for the three pheromone treatments in the first three weeks, but for weeks 4–6 and 7–10 the catch in the Isomate^®^ LBAM Plus treated plots was significantly lower than that for each of the SPLAT^™^ treatments. This was not the case for the virgin female-baited traps (Table 3), which were affected similarly in all three pheromone treatments post-treatment, offering less resolution than synthetic lures.

**Table 2:** Synthetic sex pheromone trap catch of male *Epiphyas postvittana* in three post-treatment time periods under mating disruption and lure and kill treatments in a commercial vineyard, Langhorne Creek, South Australia.

Treatment	Males per trap per day (mean ± SE)^a^		
	Weeks 1-2-3	Weeks 4-5-6	Weeks 7-8-9-10
Isomate^®^	0.0014 ± 0.0010 A	0.0057 ± 0.0019 A	0.0449 ± 0.0071 A
SPLAT^™^ mating disruption	0.0007 ± 0.0007 A	0.0221 ± 0.0061 B	0.0857 ± 0.0169 B
SPLAT^™^ with insecticide	0.0029 ± 0.0017 A	0.0307 ± 0.0102 B	0.0964 ± 0.0232 B

^***a***^ Different letters within a column indicate significant differences among treatments (*P*≤0.05; Tukey test).

**Table 3:** Trap catches of males to virgin female *Epiphyas postvittana* pre- and post-treatment with mating disruption (MD) or lure and kill (SPLAT^™^ + insecticide) treatments in a commercial vineyard, Langhorne Creek, South Australia.

Treatment	Males per trap per day (mean ± SE)^a^		% Disruptive Index
	Pre-treatment	Post-treatment	
Control	0.3929 ± 0.1001 A	0.1429 ± 0.0426 A	
Isomate^®^	0.2232 ± 0.0513 A	0.0010 ± 0.0010 B	99.3
SPLAT^™^ MD	0.1875 ± 0.0486 A	0.0010 ± 0.0010 B	99.3
SPLAT^™^ + insecticide	0.4018 ± 0.1591 A	0.0031 ± 0.0022 B	97.8

^***a***^ Different letters within a column indicate significant differences among treatments (P≤0.05; Tukey test).

Before treatment application, the number of male LBAM captured in the virgin female-baited traps was not significantly different between plots, as expected (*F*_*3*, *9*_ = 0.96; *P* = 0.452). However, for seven weeks post-treatment the male catch was significantly lower in the plots treated with a pheromone treatment than in the untreated control plots (*F*_*3*, *9*_ = 6.76; *P* = 0.0111) (Table 3).

### Experiment 2: Relative attractiveness of point sources of SPLAT^™^ HD LBAM

The synthetic two component pheromone lures were significantly (38-fold) more attractive than per individual than virgin females. The SPLAT dollops, with or without insecticide, were not significantly more attractive than five virgin females (Table 4).

**Table 4:** Trap catches of *Epiphyas postvittana* males to laboratory-reared virgin female moths, rubber septa (3 mg, two-component), and SPLAT^™^ with or without insecticide in a commercial vineyard, Langhorne Creek, South Australia. The traps (four replicates per treatment) were cleared weekly for seven weeks.

Treatment	Mean per trap	SEM		Female equivalent
SPLAT^™^ mating disruption	7.978	2.3702	a	4.5
SPLAT^™^ + insecticide	12.221	2.5409	a	7.0
Pheromone, 3 mg	68.438	1.4869	b	38
Virgin females (5)	8.8287	1.5667	a	5.0

*F*_3,15_ = 8.16, *P*<0.01.

### Experiment 3: Point-source density effect on mating disruption from SPLAT^™^ HD LBAM

In the 4 weeks prior to 22 November 2010, when the application of the SPLAT treatments at different point-source densities commenced, the mean number of male LBAM captured in the pheromone traps did not differ significantly among the treatment plots (*F*_*4*, *16*_ = 0.34; *P* = 0.850) (1.18 moths per trap per day). During the post-treatment period (24 November 2010 to 9 March 2011), the mean number (±SE) of moths captured in the pheromone traps in the control plots reduced to 0.37±0.06 (range: 0.09–0.75) per trap per day, because of seasonal phenology.

**Fig 2:**
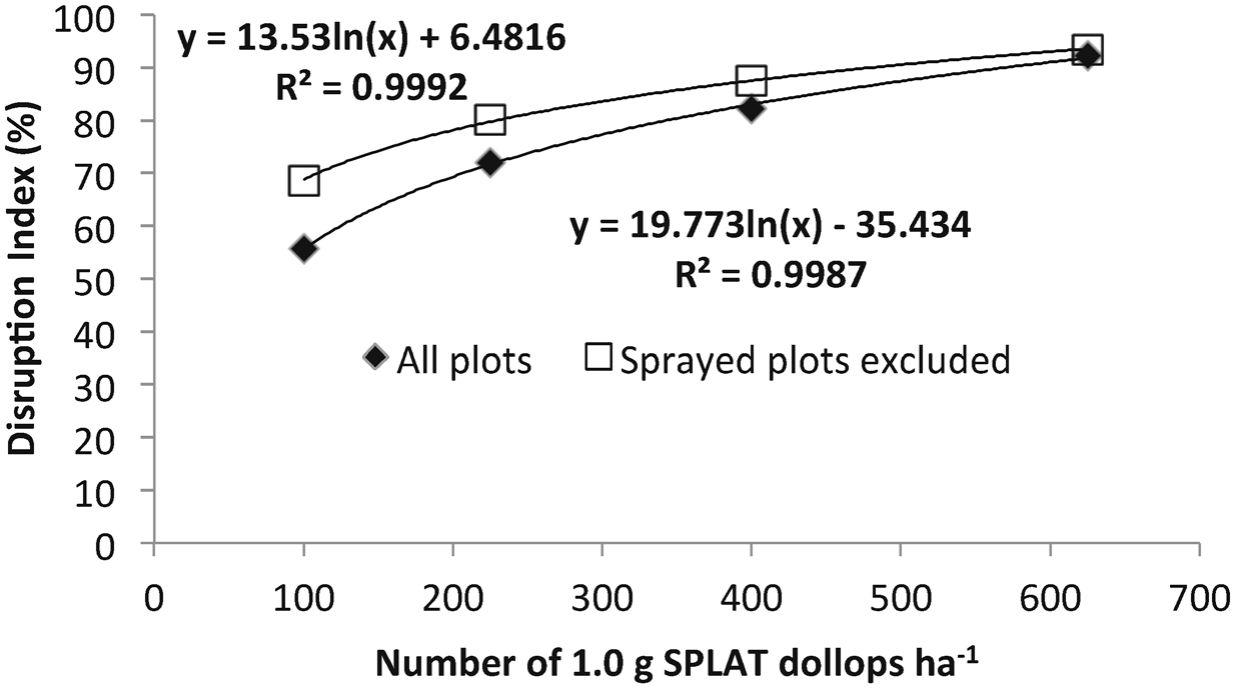
Effect of insecticide treatment of some plots on the Disruption Index of *Epiphyas postvittana*, as a function of point source density of pheromone sources. Removal of sprayed plots had a limited effect only, in apparently lowering the efficacy of the pheromone.

There was a very significant negative effect of SPLAT^™^ point source density on the number of moths captured in pheromone traps (Table 5), with a strong rate response (F_4,170_ = 23.64, P<0.001). This effect was evident irrespective of the inclusion or omission of the Avatar^™^-treated plots from the analysis (Fig 2). The relationship between the degree of the Disruption Index and rate of SPLAT^™^ was well described by a logarithmic curve. The fitted models, with and without the Avatar^™^-treated plots, estimated that the achievement of a DI of 99.0% would require 897 and 933 dollops ha^-1^ respectively, and a DI of 99.9% would require 939 and 997 dollops ha^-1^ respectively.

**Table 5:** The mean of male catch of *Epiphyas postvittana* as a function of point source density of LBAM SPLAT^™^ over ten weeks in a commercial vineyard, Langhorne Creek, South Australia (five replicates, 1 ha plots).

SPLAT^™^ treatment (g ai ha^-1^)	Mean^a^	SEM
0	17.31	1.82
100	7.51	1.64
225	5.05	1.76
400	2.91	1.54
625	1.30	1.25

^*a*^ N = Back-transformed mean number of moths trapped.

### Experiment 4: Combination of insecticide and mating disruption technologies

In the three weeks prior to the Prodigy^®^ spray application on 7–8 November 2011, the mean number (±SE) of male LBAM captured in the pheromone traps was 0.45 ± 0.07 (range: 0.03–0.78) per trap per day and this did not differ significantly among the treatment plots (F_2,4_ = 3.53; *P* = 0.1310). During the 22-week period (10 November 2011 to 19 April 2012) following the Prodigy^®^ application, the mean number (±SE) of moths captured in the pheromone traps in the control plots was 0.17 ± 0.04 (range: 0.01–0.78) per trap per day.

**Fig 3:**
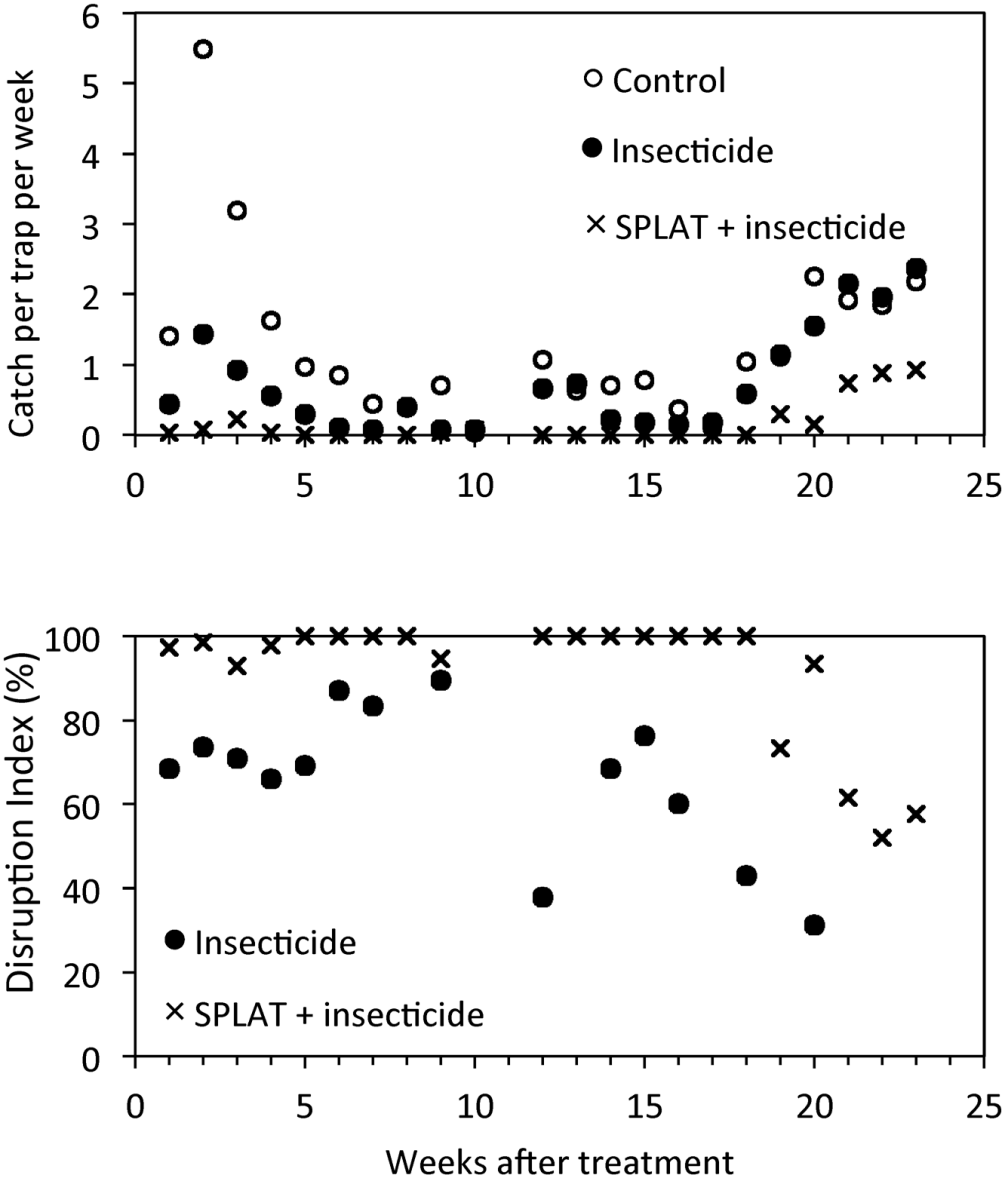
Mean weekly catch per trap of *Epiphyas postvittana* in monitoring traps in a commercial vineyard, Langhorne Creek, South Australia (top) with methoxyfenozide insecticide application made against eggs and larvae (application in week 0), comparing plots with or without SPLAT^™^ LBAM (applications in week 0 and week 11), and untreated controls (9-ha plots). The Disruption Index is also shown (bottom).

Consistent with the mode of action of methoxyfenozide, the insecticide spray treatment had no immediate effect on the numbers of LBAM moths trapped in the initial fortnight (10–24 November 2011) following application (*F*_2,4_ = 1.10; *P* = 0.4144). However, in the 10-week assessment period following the first SPLAT^™^ application on 28–29 November 2011, pheromone trap catches were significantly reduced in all insecticide treated plots compared with those in the untreated control, and the reduction was significantly greater in the plots treated with insecticide and SPLAT^™^ than with insecticide alone (F_2,4_ = 53.59; *P* = 0.0013) (Fig 3 and Table 6). In the subsequent 10-week assessment period, which followed the second SPLAT^™^ application on 13–15 February 2012, pheromone trap catches were again significantly lower in the insecticide plus SPLAT^™^ treated plots than in the control plots (F _2,4_ = 8.61; *P* = 0.0355), whereas the catches in the control plots and those treated with insecticide alone were statistically similar (Table 6).

**Table 6:** Mean catch of male *Epiphyas postvittana* in pheromone traps (male moths per plot) in two post-treatment time periods, in a combination of insecticide (methoxyfenozide) and mating disruption (SPLAT^™^ LBAM) experiment in a commercial vineyard, Langhorne Creek, South Australia.

Treatment	Weeks 1–10	Weeks 11–20
	Mean ± SE^a^	Mean ± SE^a^
Control	136.0 ± 47.1 A	72.3 ± 35.5 A
Methoxyfenozide alone	39.7 ± 8.4 B	49.0 ± 5.7 AB
Methoxyfenozide + SPLAT^™^	4.3 ± 1.5 C	4.0 ± 2.1 B

^*a*^ Different letters within a column indicate significant differences among treatments (P≤0.05; Tukey test).

The mating rates of virgin female moths (n = 12 per treatment over 17 weeks) were 10% in the control (19 females), 5% in the insecticide treatment (10 females) and 1% (2 females) in the combination of mating disruption and insecticide. The low values in controls (10% mating) limit the value of these data, although they trend in agreement with traps baited with synthetic lures.

The insecticide-SPLAT^™^ treatment resulted in mean DI values of 97.0% and 90.7% in the 10 weeks following the first and second SPLAT treatments respectively (Table 7). In the eighth week following the second SPLAT^™^ application, two days of high winds occurred, which may have contributed to the marked decline in the DI value from the previous seven weeks’ value of 100%, to 60.4%, although the values remained low thereafter. Comparison of the estimated and observed suppression form the combination of pheromone at 625 points/ha and insecticide spray application gave close alignment (within 1%) with the additive model for activity (Table 7).

**Table 7:** Comparison of models of interaction between mating disruption and insecticide treatments in 9-ha vineyard plots treated with mating disruption and insecticide (Prodigy^®^), alone and together, to test whether the interaction was additive or synergistic.

Tactic	Suppression Gen 1	Suppression Gen 2	Source
Mating disruption (625 points/ha)	93.5%	93.5%	Expt 3, Table 3
Prodigy^®^ (one application)	70%	30%	Expt 4
Prodigy + SPLAT^™^ (two applications)	97.1%	95.5%	Expt 4
Expected (No interaction, additive)	98.0%	95.4%	Expt 3 and 4

Data have been supplied (S1–S6 Tables).

## Discussion

Barclay et al. [41] showed how complementarity between tactics could be examined by plotting effort-mortality curves, but these data are difficult to obtain, and few have attempted this approach. Boukal and Berec [42] explored interactions between tactics from the perspective of interaction between Allee effects which can result in raising of the Allee threshold higher than the sum of the thresholds for individual tactics, and a bioeconomic perspective can also be taken, with synergy defined by cost savings from the combination being greater than for single tactics alone [9].

We have used another approach here, together with field estimates for tactics from field trials obtained from a series of inter-related experiments. Our hypothesis was that the expected inverse density dependence of the pheromone treatment might lead to synergy of tactics, which could be estimated by the product of the suppression from two tactics (additive), or increased further as reflected in a significant (positive) interaction term.

The potential of SPLAT^™^ LBAM HD pheromone formulations (ISCA Technologies, Riverside) for suppression of LBAM has been demonstrated in three Australian vineyard experiments, where it compared favourably with the polythethylene tubing product developed previously. It is acknowledged that all results are based on males caught, not larval populations. In the first experiment, all the pheromone formulations tested achieved similar high rates of disruption of the male LBAM population, as measured by both pheromone and virgin female trap catches for the 10 week post-treatment assessment period.

The degree of disruption provided by the Isomate^®^ treatment persisted for longer than that of the SPLAT^™^ treatments; for example, during the final four weeks of post-treatment assessment the disruption to the pheromone trap catch by these three pheromone treatments was 94.7%, 89.8% and 88.5% respectively. However, because the rates of application of the two major LBAM pheromone components in the SPLAT^™^ MD and SPLAT^™^ plus insecticide lure and kill treatment were lower (respectively 27.1% and 30.6% lower than that applied with the Isomate^®^ treatment), this difference is likely to have been a primary contributor to the lesser effect and persistence of the disruptive effect of the SPLAT^™^ treatments relative to that of the Isomate^®^ ties.

Given the similar performance of the two SPLAT^™^ HD LBAM formulations in the first experiment, the subsequent studies only used the mating disruption formulation, because the potential to use a formulation without an insecticide would be expected to have broader community acceptance and easier registration. The lack of a difference in performance between lure and kill and disruption formulations was surprising, since this has been recorded before and could be expected if males were being killed [28,29]. A reduced level of attraction to the dollops (compared to septa) probably explains the effect here, and could be caused by pheromone impurities. This underscores the need to understand behavioral mechanisms.

Furthermore, it needs to be taken into account that in a comparison of the relative attraction of traps loaded with either five virgin LBAM female moths or 3 mg LBAM two-component pheromone septa, the pheromone blend was more than 38 times as attractive as single virgin females would be expected to (Table 4). This raises the possibility that the calculated disruption efficacy referenced in these experiments may be an underestimate of the disruption to female attraction (false negative), since it is more difficult to disrupt attraction to stronger point sources. Hence less than complete disruption of synthetic sources could yield population suppression in practice, providing a conservative estimate of effect. The use of a laboratory colony could have contributed to low male catches to virgin females, although the pheromone titre in this colony was found to be in the normal range (Suckling et al. unpublished data).

In the third field experiment, a strong rate response with SPLAT^™^ HD LBAM occurred, consistent with the results of Suckling et al. [21] in New Zealand, where the top density was 500/ha. The degree of disruption to the synthetic pheromone trap catch at the highest rate (625 g ha^-1^) of SPLAT^™^ alone was 93.5%. By extrapolation of the fitted model, a rate of between 900 and 1,000 1.0 g dollops ha^-1^ would be likely to be required to achieve a 99.0% to 99.9% Disruption Index. There was an effect limited to low point source densities from the unplanned insecticide. The use of two-component pheromone here suggests that improvements could be made in efficacy by blend improvements, adding the two minor components recently reported since the four component blend is more attractive [43]. The specific effects of these compounds as components of communication disruption have been examined elsewhere [20].

The disruptive effect of the tested SPLAT formulations generally persisted over the ten-week test period post-application in each of the three field experiments. This is an encouraging result, given that the prevailing weather conditions were generally warm to hot. For example, the mean daily maxima in Experiments 2 and 3 exceeded 26°C, and during both experiments 40–42°C heat-waves occurred in South Australia. The longevity of disruption reflects the performance seen in cooler New Zealand studies [23].

The capacity to mechanize the ground and aerial applications of SPLAT^™^ means that the treatment of extensive areas of LBAM host crop and surrounds using this technology is feasible at potentially lesser cost, treatment time and dependence on local landscape features compared with the standard polyethylene tubing technology. These features make SPLAT^™^ HD a potentially valuable technology for large-scale mating disruption of an emergency plant pest incursion, which could be deployed either alone or in combination with other compatible tactics.

These results demonstrate that a substantial disruption of male moth catch can be achieved by the 625 g a.i. ha^-1^ rate of SPLAT^™^, and that deployment at this rate has potential as a combination tactic for emergency plant pest eradication. The lower rates tested had lesser disruptive effects, but higher rates could provide a cost-effective pest management tool as an alternative to insecticidal control of LBAM.

The ‘combination’ treatment of the registered rate of the insecticide Prodigy^®^ followed by the MD treatment with SPLAT LBAM HD appears to have provided a very substantial suppression of LBAM adult activity, as measured by pheromone trapping. However, a comparison of the rates of suppression seen in both generations with the calculated expected suppression from an additive interaction (mathematical product of survivorships for each tactic), suggests that this model is explains the results. Synergistic effects could be expected to have much greater suppression in the combination, because of an interaction term.

The duration and degree of disruption observed in the final experiment appears similar to those observed in earlier experiments. SPLAT^™^ HD LBAM applied at a rate of around 625 g ai ha^-1^ as approximately 1.0 g dollops provided effective communication disruption (and presumed mating disruption) for about 10 weeks, and then this effect appears to decline significantly. For 7 or 8 weeks, the results were close to 100% disruption each week.

The potential was assessed to suppress a field population of LBAM to near localized extinction using SPLAT^™^ LBAM HD applied in combination with a foliar insecticide treatment. If competitive attraction is the mechanism responsible for the mating disruption effect,[10] then the success of mating disruption as a pest management or eradication tactic would be expected to be dependent on the pest density, and likely to require deployment in combination with another tactic (e.g. insecticidal control) to ensure the pest population density is sufficiently low for mating disruption to succeed.

Based on the trial results to date and the capacity to mechanize the application of SPLAT^™^, the treatment of extensive areas of LBAM host crop and surrounds using this technology is feasible at lower cost, treatment time and dependence on local landscape features than the standard polyethylene tubing technology. These features make SPLAT^™^ a potentially superior technology than existing pheromone products in Australia for large-scale mating disruption of an emergency plant pest incursion.

These technologies are very selective, with minimal non-target effects, and may be highly efficacious for pest suppression or eradication when deployed either alone or in combination with other compatible insect-control technologies, such as selective insecticides and the sterile insect technique. However, areawide treatment is need to gain longer-term benefits of pest suppression over the opposing effect of re-immigration.

Work on the effects of multiple tactics has been seldom undertaken in pest management in part because of the resource requirements for combination treatments at field scale, and a lack of motivation to separate effects from expensive treatments that are not normally combined in practice in commercial pest management, where a higher residual population is tolerated than during an eradication [6]. More cases of practical evaluation are needed to better understand how synergistic results may be achieved between pest suppression tactics, particularly for use in eradication programs.

## Supporting Information

S1 Table 2009 Experiment 1 (male catch to septa lures, data). (XLSX)

S2 Table 2009 Experiment 1 (male catch to 3 females, data). (XLSX)

S3 Table 2010 Experiment 2 (male catch to various lures, data). (XLSX)

S4 Table 2010 Experiment 3 (male catch to septa lures, data). (XLSX)

S5 Table 2011 Experiment 4 (male catch to septa lures, data). (XLSX)

S6 Table 2011 Experiment 4 (single female mating, data). (XLSX)
